# Imaging the Polymorphic Transformation in a Single Cu_6_Sn_5_ Grain in a Solder Joint

**DOI:** 10.3390/ma11112229

**Published:** 2018-11-09

**Authors:** Flora Somidin, Hiroshi Maeno, Xuan Quy Tran, Stuart D. McDonald, Mohd Arif Anuar Mohd Salleh, Syo Matsumura, Kazuhiro Nogita

**Affiliations:** 1 Nihon Superior Centre for the Manufacture of Electronic Materials (NS CMEM), School of Mechanical and Mining Engineering, The University of Queensland, Brisbane QLD 4072, Australia; s.mcdonald1@uq.edu.au (S.D.M.); k.nogita@uq.edu.au (K.N.); 2 Centre of Excellence Geopolymer and Green Technology, School of Materials Engineering, Universiti Malaysia Perlis, Taman Muhibbah, Arau 02600, Malaysia; arifanuarsalleh@gmail.com; 3 The Ultramicroscopy Research Center, Kyushu University, Fukuoka 819-0395, Japan; maeno@hvem.kyushu-u.ac.jp (H.M.); syo@nucl.kyushu-u.ac.jp (S.M.); 4 Department of Applied Quantum Physics and Nuclear Engineering, Kyushu University, Fukuoka 819-0395, Japan; xuan.tran@nucl.kyushu-u.ac.jp

**Keywords:** Cu_6_Sn_5_ intermetallic compound, high-voltage transmission electron microscopy, polymorphic phase transformation, electron diffraction, time-temperature transformation

## Abstract

In-situ observations of the polymorphic transformation in a single targeted Cu_6_Sn_5_ grain constrained between Sn-0.7 wt % Cu solder and Cu-Cu_3_Sn phases and the associated structural evolution during a solid-state thermal cycle were achieved via a high-voltage transmission electron microscope (HV-TEM) technique. Here, we show that the monoclinic η′-Cu_6_Sn_5_ superlattice reflections appear in the hexagonal η-Cu_6_Sn_5_ diffraction pattern upon cooling to isothermal 140 °C from 210 °C. The in-situ real space imaging shows that the η′-Cu_6_Sn_5_ contrast pattern is initiated at the grain boundary. This method demonstrates a new approach for further understanding the polymorphic transformation behavior on a real solder joint.

## 1. Introduction

The intermetallic Cu_6_Sn_5_ is a critical compound that typically forms in a layer between Sn-rich solder alloys and Cu substrates in printed circuit boards (PCB) of electronic packaging systems and has potential as an advanced anode material for Li-ion batteries [1,2]. It is well known that Cu_6_Sn_5_ undergoes a polymorphic transformation from hexagonal η-Cu_6_Sn_5_ (P6_3_/mmc) above 186 °C and transforms to monoclinic η′-Cu_6_Sn_5_ (C12/c1) at lower temperatures [3,4]. A typical lead-free solder joint contains a variety of phases in intimate contact with one another, such as Cu, Cu_3_Sn, Cu_6_Sn_5_ and Sn and is constantly subjected to thermal cycling during service. As a result, the joint is vulnerable to the 2.15% volume change that is associated with the η ↔ η′-Cu_6_Sn_5_ transformation, which may lead to mechanical failure [5,6,7].

Previous studies have shown that the additions of alloying elements such as Ni, Zn and Au [6,8,9] into the Cu_6_Sn_5_ intermetallic compound (IMC) can stabilise the high temperature hexagonal phase and prevent transformation. In fact, the Sn-Cu-Ni solder alloy system has been favourably used in the interconnect industry as the (Cu, Ni)_6_Sn_5_ intermetallic compounds (IMCs) formed between the solder alloy and Cu substrates have been frequently reported to demonstrate a better joint stability, when compared to the Cu_6_Sn_5_ IMC [5,6,9,10,11,12,13]. In stoichiometric samples, it has been shown that the Cu_6_Sn_5_ IMC has higher coefficient of thermal expansion (CTE) values than (Cu, Ni)_6_Sn_5_ [8]. It has been reported that less-apparent cracks were found in the interfacial Cu_6_Sn_5_ layer when Ni is present in the solder joint [6]. However, the expected change in volume associated with the polymorphic transformation can be varied in the real solder joint, with the continuous presence of Sn and Cu migration into the Cu_6_Sn_5_ intermetallic compound (IMC) layer during the transformation. Moreover, there is no clear consensus regarding the mechanisms by which the local transformations within a single-Cu_6_Sn_5_ grain in a real solder joint could instigate cracking in the IMC layer. Thus, it is important to understand the significance of the polymorphic transformation in more commercially relevant structures.

It has been suggested that the polymorphic transformation that occurs in Cu_6_Sn_5_ present in real solder joint is of significance for reliability; however, characterising this transformation is technically challenging [14,15]. The kinetics of polymorphic transformation in Cu_6_Sn_5_ were extensively investigated using X-ray diffraction (XRD) methods [10,16,17] and differential scanning calorimetry (DSC) [7,14]. However, these investigations were conducted in single-phase powdered samples without considering the contribution and significance of the Cu_6_Sn_5_ IMC surrounding phases (i.e., Cu, Cu_3_Sn and Sn) that are present in a real solder joint. In our recent work, the time-temperature-transformation (TTT) of Cu_6_Sn_5_ was constructed using data obtained from high-voltage transmission electron microscopy (HV-TEM) [18] on a single-targeted Cu_6_Sn_5_ grain, which is intimately attached to both Cu_3_Sn and Sn-rich areas. It was observed that the transformation can be controlled during soldering and the hexagonal phases remain stable at room temperatures if the rates of cooling exceeded 20 °C·min^−1^ when cooling from 210 °C. The transformation from hexagonal to monoclinic was fastest in the temperature range of 135 to 150 °C. In the current work, we used this previously-established TTT curve and systematically observed the effect of the polymorphic transformation in real-space imaging in a single-Cu_6_Sn_5_ grain at temperatures associated with the fastest rate of transformation. The results are an important progression in the understanding of transformations in industrially relevant solder joints.

## 2. Materials and Methods 

**Figure 1:**
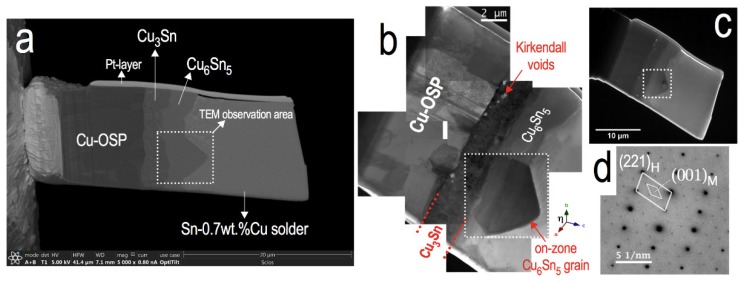
FIB lamella sample. (**a**) Backscattered SEM image; (**b**) high and (**c**) low magnification bright-field plasmon-filtered TEM images; (**d**) SADP of the fresh selected Cu_6_Sn_5_ grain at room temperature before the in-situ heating experiment.

In this study, a TEM lamella sample from a solder joint consisting of Cu/Cu_3_Sn/Cu_6_Sn_5_/Sn-rich interfaces was prepared using a focused ion beam (FIB) system (FEI Scios Dual-beam FIB, Thermo Fisher Scientific Waltham, MA, USA) as shown in Figure 1a. The sample was extracted from an annealed solder joint between a Sn-0.7 wt % Cu solder ball of diameter 0.6 mm (Nihon Superior Co. Ltd., Osaka, Japan) and a Cu substrate printed circuit board (PCB) with an organic soldering preservative (OSP) surface finish. The solder joint sample was first assembled by reflow soldering and subsequently annealed for 500 h at 150 °C to produce a thicker IMC layer. The FIB-prepared sample (as shown in Figure 1a) thickness was about 0.5 μm, which is nearly 5 times thicker than that observable using conventional TEM. Not only is the thick TEM specimen more representative of the bulk solder, but it also provides more structural rigidity, which is important for characterising a metastable sample when subjected to multiple heating cycles. The thick lamella sample (Figure 1a) was characterised using a JEM-1300NEF (JEOL, Akishima, Japan) at an accelerating voltage of 1250 kV. This HV-TEM at the Research Laboratory for High Voltage Electron Microscopy, Kyushu University, is equipped with an in-column omega-type energy filter and this has the advantage of allowing thicker specimens to be imaged at higher contrast [19,20]. The beam induced heating on Cu_6_Sn_5_ is about 0.5 times less (see Appendix A) in the case of high voltage compared to conventional low voltage TEM, allowing for proper observations on the metastable sample. The lamella sample was placed on a double-tilt heating holder (EM-HSTH JEOL, Akishima, Japan) and on-zone bright-field TEM images of the sample were obtained (Figure 1b,c).

To observe the phase transformation in diffraction mode, the TEM sample was first heated to 210 °C at a heating rate of approximately 33.6 °C·min^−1^ from room temperature, and SADPs in the same zone condition were obtained. The sample was then cooled down to 140 °C by turning off and resetting the heater to this temperature. A series of SADPs from the same area of the Cu_6_Sn_5_ grain was captured at 60 s intervals as the temperature stabilised to 140 °C. The camera length and exposure time were fixed at 679.62 mm and 30 s for each captured SADP, respectively. In this study, all obtained SADPs were analyzed using the Gatan Digital Micrograph software (v2.32, Gatan, Inc., CA, USA) and Single Crystal software (Crystal Maker, v3.0, CrystalMaker Software Ltd., Oxford, UK), and presented in reverse grey-scale colour map for better visibility. 

## 3. Results and Discussion

**Figure 2:**
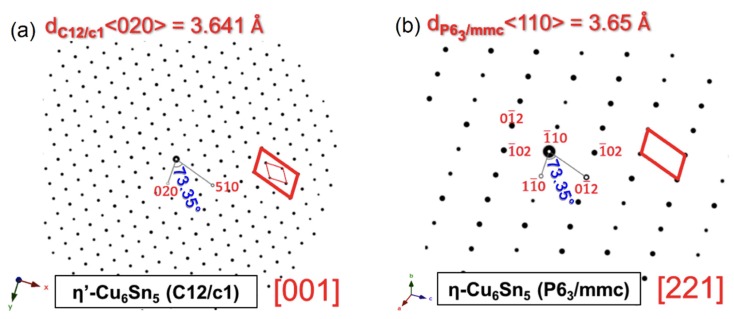
Simulated electron diffraction patterns of the (**a**) monoclinic η′-Cu_6_Sn_5_ C12/c1 structure and (**b**) hexagonal η-Cu_6_Sn_5_ P6_3_/mmc structure corresponding to the obtained SADPs in this study.

The crystal structure of the chosen Cu_6_Sn_5_ grain at room temperature (RT) was identified as η + η′-Cu_6_Sn_5_ based on a selected area diffraction pattern (SADP) shown in Figure 1d. The hexagonal η-Cu_6_Sn_5_ is in the (221) plane and the weaker reflections can be indexed as monoclinic η′-Cu_6_Sn_5_ in the (001) plane. The simulated patterns are shown in Figure 2. The occurrence of weak monoclinic spots in the fresh Cu_6_Sn_5_ grain was expected as the solder joint sample had been annealed at 150 °C for 500 h. The presence of Kirkendall voids at the relatively thick Cu_3_Sn layer, as shown in Figure 1b, is the result of the lengthy annealing time [21,22,23]. According to the TTT diagram of Cu_6_Sn_5_ under these annealing conditions, the formation of the monoclinic phase is likely to be retained when cooled from 150 °C to RT [18].

**Figure 3:**
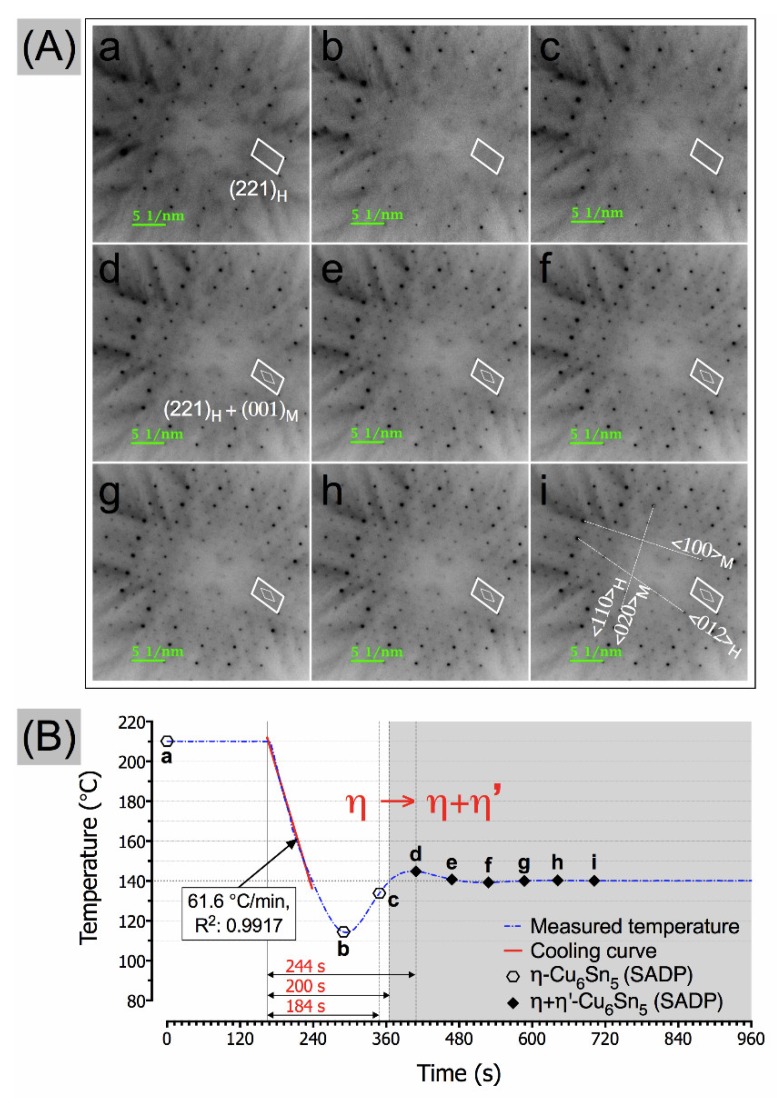
(**A**) Corresponding SADPs in the observed Cu_6_Sn_5_ grain from time point-a to -i in the (**B**) profile temperature of the in-situ HV-TEM observations in diffraction mode.

The corresponding SADPs and the temperature profile of the cooling experiment are shown in Figure 3. The average cooling rate was 61.6 °C·min^−1^, which was estimated from the cooling curve in the temperature profile. At the isothermal condition of 210 °C (time point-a in Figure 3), only the η-Cu_6_Sn_5_ reflections were observed in the captured SADP. This is expected as the hexagonal η-Cu_6_Sn_5_ (P6_3_/mmc) phase exists above 186 °C [3,24]. As the sample isothermally stabilised at 140 °C, weak monoclinic spots (indicated as diamond shape box) were eventually observable in the captured SADPs starting at time point-d, and gradually became more distinguishable. The onset of the η → η + η′-Cu_6_Sn_5_ transformation is believed somewhere between time point-c and -d, which is around >184 to ≤244 s after being cooled from 210 °C based on the corresponding SADPs in Figure 3. The polymorphic η to η + η′-Cu_6_Sn_5_ transformation behaviour observed in this study is in good agreement with our previously reported results, in which the first sign of discernible η′-Cu_6_Sn_5_ reflections in the captured SADPs in a single Cu_6_Sn_5_ grain when cooled from 210 °C to between 150 °C and 135 °C occurred around >195 to <263 s [18].

The transformation in the SADP is a sign of modification of the atomic arrangements and could possibly be observed through high resolution TEM imaging techniques [25]. Because of the thick sample we used, atomic resolution changes that corresponded to the transformation in the SADPs on the same sample in this current experiment were not observed. Thus, the morphological changes in Cu_6_Sn_5_ in this study was observed through low and high magnification bright-field TEM imaging. However, any deformation causes tilting of the local crystal lattice and this may arise indirectly through a polymorphic transformation of the Cu_6_Sn_5_ phase that is constrained by adjacent material. Such deformation may manifest as bend contour movements. Nogita et al. [26] have shown evidence of stress creation and release events by imaging bend contours and the associated volumetric change in a Cu_6_Sn_5_ grain obtained from an annealed Sn-0.7 wt % Cu/Cu solder joint during in-situ heating from RT to 210 °C under HV-TEM. They reported bend contours movements started at around 180 °C, which correspond to the monoclinic to hexagonal polymorphic transformations of the Cu_6_Sn_5_ phase [26].

**Figure 4:**
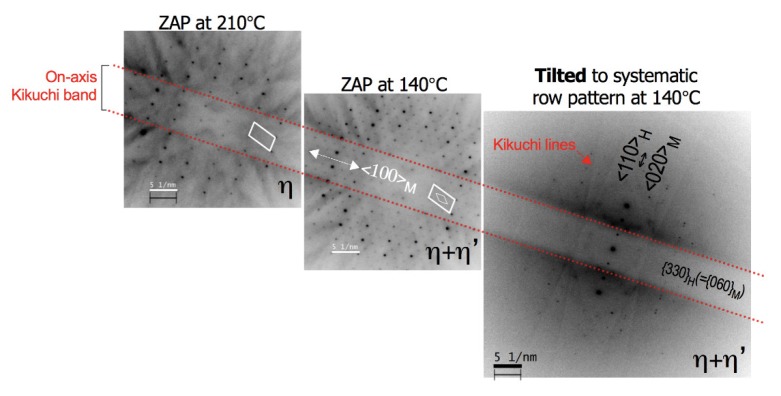
Series of SADPs in this study tilted to the on-axis {330}_H_ (={060}_M_) Kikuchi band.

In this experiment, we only considered diffraction contrast effects from a single row of reflections of known zone axis to avoid complexity of contrast interpretation. Before the second heating/cooling cycle is carried out, the zone-axis-pattern (ZAP) as in Figure 3i was first tilted to a few degrees away (in a <100>_M_ direction) from [221]_H_ (=[001]_M_) zone axis to obtain the <110>_H_ (=<020>_M_) on-axis systematic row pattern, as shown in Figure 4, at isothermal condition of 140 °C. The Kikuchi lines can be seen in the diffraction pattern image of a sample with ideal thickness under HV-TEM.

**Figure 5:**
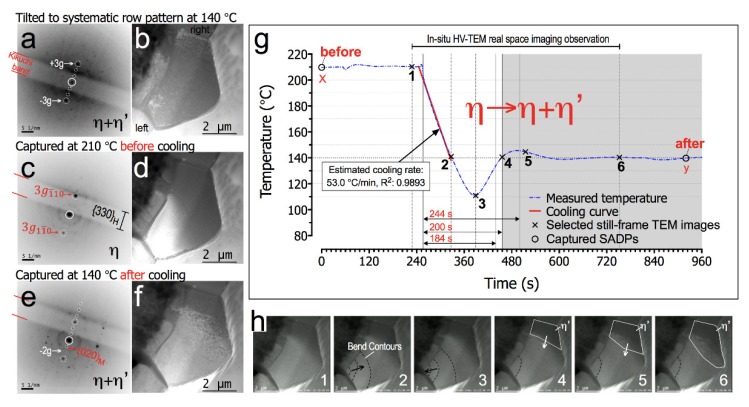
(**a**,**c**,**e**) bright-field plasmon-filtered TEM images corresponding to captured SADPs in (**b**,**d**,**f**); (**g**) Temperature profile of the in-situ HV-TEM observations; (**h**) selected still-frame TEM images from in-situ video taken at time points 1 to 6 in the temperature profile showing the observation analysis. Video of the in-situ cooling observation can be seen in Supplementary Video S1.

Note that the ±3 g reflections in Figure 5a are strongly excited. The sample was then heated up to 210 °C and the corresponding SADP captured at point-x in temperature profile of Figure 5g is shown in Figure 5c. Interestingly, the weak reflections that are previously observed in Figure 5a (±1 g and ±2 g spots) have disappeared. These weak reflections then high likely belong to the η′-Cu_6_Sn_5_ set of planes_._ The {330}_H_ Kikuchi band has slightly moved and passed the diffracted +3 g spot, intensifying its reflection. The right side of the Cu_6_Sn_5_ grain also appeared darker in the bright-field image (see Figure 5d). This Kikuchi band movement was likely caused by a slight change in the on-axis tilt condition due to volume change in the Cu_6_Sn_5_ grain. This behavior can be attributed to the η + η′-Cu_6_Sn_5_ to η transformation during heating from an isothermal condition of 140 to 210 °C. To investigate the η to η + η′-Cu_6_Sn_5_ transformation effect on the Cu/Cu_3_Sn/Cu_6_Sn_5_/Sn-rich interfaces during cooling, in-situ bright-field HV-TEM observations were performed using similar cooling experiment conditions as in Figure 3 and the event was recorded using a high-resolution video recorder.

The temperature profile for the bright-field diffraction contrast during in-situ HV-TEM observation and the analysis of the contrast patterns behaviour are shown in Figure 5g,h, respectively. From time point-1 to point-2 (see Supplementary Video S1), the thick FIB sample was quite stable without sudden large movements or tilting that could lead to obvious changes in diffraction contrast, as the sample was cooled at the rate of around 53 °C·min^−1^ from 210 to 140 °C. As shown in Figure 5h, there is bend contour movement (annotated with dashed lines) appearing at the left side of the Cu_6_Sn_5_ grain boundary, which becomes more apparent between time point-2 to point-4. A further look at the bend contours’ movement behavior between time point-2 to point-3 showed movement from the grain boundary to the center of the grain and a reversing of its direction at some time in between point-3 to point-4 (see Supplementary Video S1). As the sample stabilizes to its isothermal condition at 140 °C from time point-4 onwards, the bend contour movement starts to cease and remains stagnant until time point-6; at least within the observation timeline of this in-situ observation. At the same time, new darker contrast patterns grew at the right side of the Cu_6_Sn_5_ grain boundary (annotated with white solid lines in Figure 5h from time point-4 to point-6). 

This new contrast patterns, which have a rough dark texture, are believed to be diffraction contrast from η + η′-Cu_6_Sn_5_ based on the bright-field image in Figure 5b. The SADP of Cu_6_Sn_5_ at time point-y in the graph after the in-situ observation and its corresponding TEM image are shown in Figure 5e,f, respectively. Here, we observed the reappearance of the weak reflections of η′-Cu_6_Sn_5_ in the systematic row pattern. The Kikuchi band has moved further away from its previous position due to volume change as the hexagonal phase transformed to η + η′-Cu_6_Sn_5_ phase. This confirms that the growing rough dark texture contrast at the right side of the grain belongs to the growing {020}_M_ planes. Note the missing −2 g spot and the contrast difference in both sides of the grain shown in Figure 5f, which may suggest that some area in the left side of the grain remains in the hexagonal phase when the SADP was captured.

**Figure 6:**
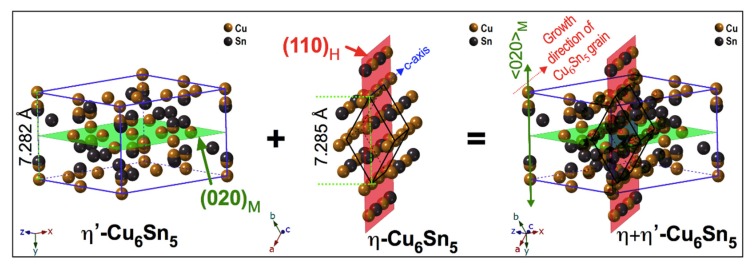
The monoclinic and hexagonal structures simulated by Single Crystal software showing the (020)_M_ and (110)_H_ planes, respectively.

It is also interesting to note that the growth directions of both (020)_M_ and (110)_H_ planes are not parallel but, intersecting, which could lead to crack formation, if the two growing phases become separated. It has been frequently reported that the Cu_6_Sn_5_ grain grows along the <001>_H_ direction. In this study, we observed that the η′-Cu_6_Sn_5_ phase grows along the <020>_M_. The simulated η + η′-Cu_6_Sn_5_ structure shows that both (020)_M_ and (110)_H_ planes intersect each other, as shown in Figure 6. Based on the in-situ HV-TEM observation analysis, the onset of the η → η + η′-Cu_6_Sn_5_ transformation lies around time point-4, which is at around 200 s after being cooled from 210 °C, as shown in the temperature profile of Figure 5g. Interestingly, this result is within the expected timeline (>184 to ≤244 s) for the onset of the η → η + η′-Cu_6_Sn_5_ transformation based on the corresponding SADPs transformation analysis in Figure 3. Based on these observations, the possible nucleation mechanism of the polymorphic transformation of Cu_6_Sn_5_ in real solder joint can be described as heterogeneous nucleation of the η′ phase from a favourable facet of the original η phase at the Cu_6_Sn_5_ grain boundaries.

**Figure 7:**
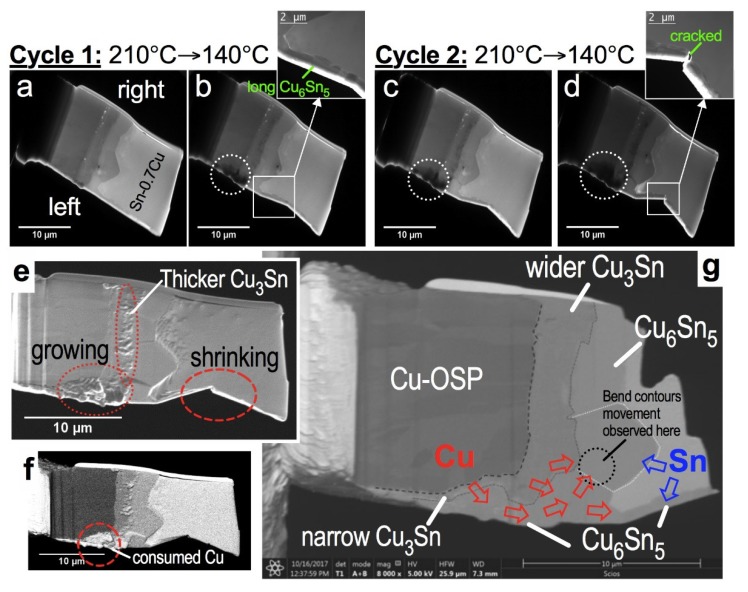
The morphology evolution of sample. (**a**–**d**) Low magnification bright-field plasmon-filtered TEM images; (**e**) secondary and (**f**,**g**) backscattered SEM images of the lamella sample after the in-situ experiment; (**g**) analysis of post-thinned FIB lamella sample.

The morphology and associated phase evolution of the FIB sample containing Cu/Cu_3_Sn/Cu_6_Sn_5_/ Sn-0.7 wt % Cu solder alloy structure in this study is shown in Figure 7. Based on the low-magnification bright-field TEM images, we notice an increasing dark contrast area in the left side of the FIB sample (encircled by dotted white circles shown in Figure 7b–d) and a significant reduction of Sn-0.7 wt % Cu area as the sample underwent multiple heating cycles. The consumption of Sn is verified with the shrinking of the Sn-0.7 wt % Cu solder area at the left side of the FIB sample, which ultimately led to the breakage of the long Cu_6_Sn_5_ grain (see insert in Figure 7d). To confirm the phase evolution in the IMC layer after the experiments, we further investigated the FIB sample under SEM. In Figure 7e,f, we can see that Cu_3_Sn thickness has significantly grown based on the region (annotated within dotted ovals) at the boundary between Cu_6_Sn_5_ and Cu_3_Sn. As seen in the backscattered SEM image in Figure 7f, the area marked by the dashed red circles does not belong to the Cu phase. To uncover the phase and structure of this unknown area, we performed post-mortem analysis using a combined SEM/FIB technique. As shown in the post-thinned sample in Figure 7g, the initial Cu area has transformed into a Cu_3_Sn/Cu_6_Sn_5_ IMC layer. Note that the Cu/Sn migration and interdifussion with a Cu_3_Sn/Cu_6_Sn_5_ layer is more conspicuous at the left side of the FIB sample. This can be attributed to the presence of a long Cu_6_Sn_5_ grain at the left side of the FIB sample, which could have accommodated most of the incoming Cu atoms (depicted as red and blue arrows in Figure 7g) from the Cu-OSP region. As a result, the Cu_6_Sn_5_ grain at the utmost left of the FIB sample has significantly grown, compared to others during the multiple in-situ heating/cooling experiments in this study. In a solder joint, where the IMC layer is continuously interacting with Sn and Cu atoms, there is contribution of interfacial reaction to the formation of the monoclinic phase, apart from the polymorphic transformation in the existing hexagonal phase within the Cu_6_Sn_5_ grain.

## 4. Conclusions

In conclusion, we examined the polymorphic transformation in a single-targeted Cu_6_Sn_5_ grain while directly observing its associated structural evolution under solid-state thermal cycling with the presence of Sn-rich and Cu-Cu_3_Sn sites, using in-situ HV-TEM observations in diffraction mode and real space imaging. The presence of a monoclinic structure in a single hexagonal η-Cu_6_Sn_5_ grain in a solder joint occurred between >184 to ≤244 s when isothermally held at 140 °C after being cooled from 210 °C, based on the appearance of weak superlattice reflections in the SADPs. The change in contrast patterns was observed at around 200 s in the in-situ HV-TEM real space imaging video. The growing monoclinic η′-Cu_6_Sn_5_ contrast pattern was found to initiate at the grain boundary on the right side of the FIB sample, while, the bend contours movement was observed on the left side the Cu_6_Sn_5_ grain, where the Cu/Sn migration and interdifussion contributions were more significant. As the interfacial reaction behaviour in a single-targeted Cu_6_Sn_5_ grain can be dependent on its surrounding phases, the effect of the polymorphic transformation might be individually unique for each Cu_6_Sn_5_ grain in the solder joint. The results reported in this work provide a general perspective on the nature of the polymorphic transformation in a single-Cu_6_Sn_5_ grain in a real solder joint, when subjected to multiple heating cycles.

## Supplementary Materials

The following are available online at http://www.mdpi.com/1996-1944/11/11/2229/s1, Video S1: In-situ HV-TEM observations in real-space imaging of the hexagonal to monoclinic transformation in single Cu_6_Sn_5_ grain on real solder joint. The video playback speed is eight times faster than normal speed.

## Appendix A

Appendix A. Estimation of inelastic electron beam-sample interaction. The following is available online at https://ars.els-cdn.com/content/image/1-s2.0-S1044580317329133-mmc1.pdf.
